# Female urethral dilatation (bougierung): a case report

**DOI:** 10.1186/s13256-018-1900-z

**Published:** 2018-12-19

**Authors:** Balint Farkas, Miklos Szakacs

**Affiliations:** 1 Department of Obstetrics and Gynecology, University of Pecs School of Medicine, 17 Edesanyak Str., Pecs, H-7624 Hungary; 2 Vivantes Humboldt Clinic, Pelvic Floor and Incontinence Centre, Am Nordgraben 2., 13509 Berlin, Germany; 3 MTA-PTE Human Reproduction Scientific Research Group, Hungarian Academy of Sciences (MTA), Pécs, Hungary

**Keywords:** Primary bladder neck obstruction, Urethral dilatation, Bougierung, Bladder outlet obstruction

## Abstract

**Background:**

Primary bladder neck obstruction is a rare clinical entity, reported to be responsible for 2.7–8% of lower urinary tract symptoms. It can lead to various urinary storage and voiding symptoms. The mainstay of treatment of female urethral strictures is urethral dilatation. Despite the long history of this method, it is unclear how far the female urethra should be dilated in correlation with residual urine volume.

**Case presentation:**

A 79-year-old Caucasian woman presented to our institute with urgency (12–15 times/day), nocturia (3 times/night), and reoccurring urinary tract infections. A physical examination revealed no anatomical malformation in her genital organs, 150 mL post-void urine retention, and a significant narrowing in the mid-segment of the urethra (4 mm). After informed consent, our patient underwent urethral dilatation ranging from Ch9 (3 mm) to Ch39 (13 mm), and reported no symptoms at the 4-week follow-up, with no post-void residual urine.

**Conclusions:**

The relatively low (around 50%) success rate of urethral dilatation might be improved by the utilization of wider dilatators, and the relaxation of the pubourethral ligament, achieved by a gentle downward saggital push during the intervention, although long-term studies with a large number of participants are necessary to prove our hypothesis.

## Background

Primary bladder neck obstruction (PBNO) is a rare clinical entity, which can occur both in men and women, and is described as the bladder neck failing to open adequately during voiding, leading to increased striated sphincter activity or obstruction of urinary flow in the absence of another anatomic obstruction, like genitourinary prolapse in women [1]. It is a relatively uncommon reason of lower urinary tract symptoms (LUTS), causing storage symptoms (frequency, urgency, urge incontinence, nocturia) and voiding symptoms (decreased force of stream, hesitancy, incomplete emptying), although in this condition the variety of symptoms may be present simultaneously in several combinations. Although the true prevalence of PBNO is not clear and little is known about the etiology, previous urinary tract infections (UTIs), trauma, or prior surgeries of the urethra are suggested to play a role in the pathogenesis of the condition [2]. The diagnosis is based on urodynamic findings, characterized by relative high-pressure, low-flow voiding (cut points for the obstruction of 15 mL/s or less for maximum flow rate [Q_max_] and greater than 20 cm H_2_O for detrusor pressure at maximum flow), as described by Nitti *et al.* [3];, however, radiographic evidence of obstruction at the bladder neck with relaxation of the striated sphincter and no evidence of distal obstruction is also necessary [1]. For its management, several approaches exist, although to date there has been no consensus as to the best care. Among the surgical modalities, urethral dilatation is considered to be the traditional method, although there is no available guideline on how wide the urethra should be dilated in correlation with urethral stricture level, or residual urine volume. Despite the majority of the available literature reporting dilatation until charrier (Ch) 30 (10 mm), we report a case of a patient whose symptomatic urinary retention (150 ml for 1 year) was successfully eliminated by urethral dilatation of Ch 39 (13 mm), without urethral injury. The following case report is rare, as it provides information about an advanced degree of urethral dilation together with successful elimination of residual urine and no urethral injury in the long term.

## Case presentation

**Fig. 1:**
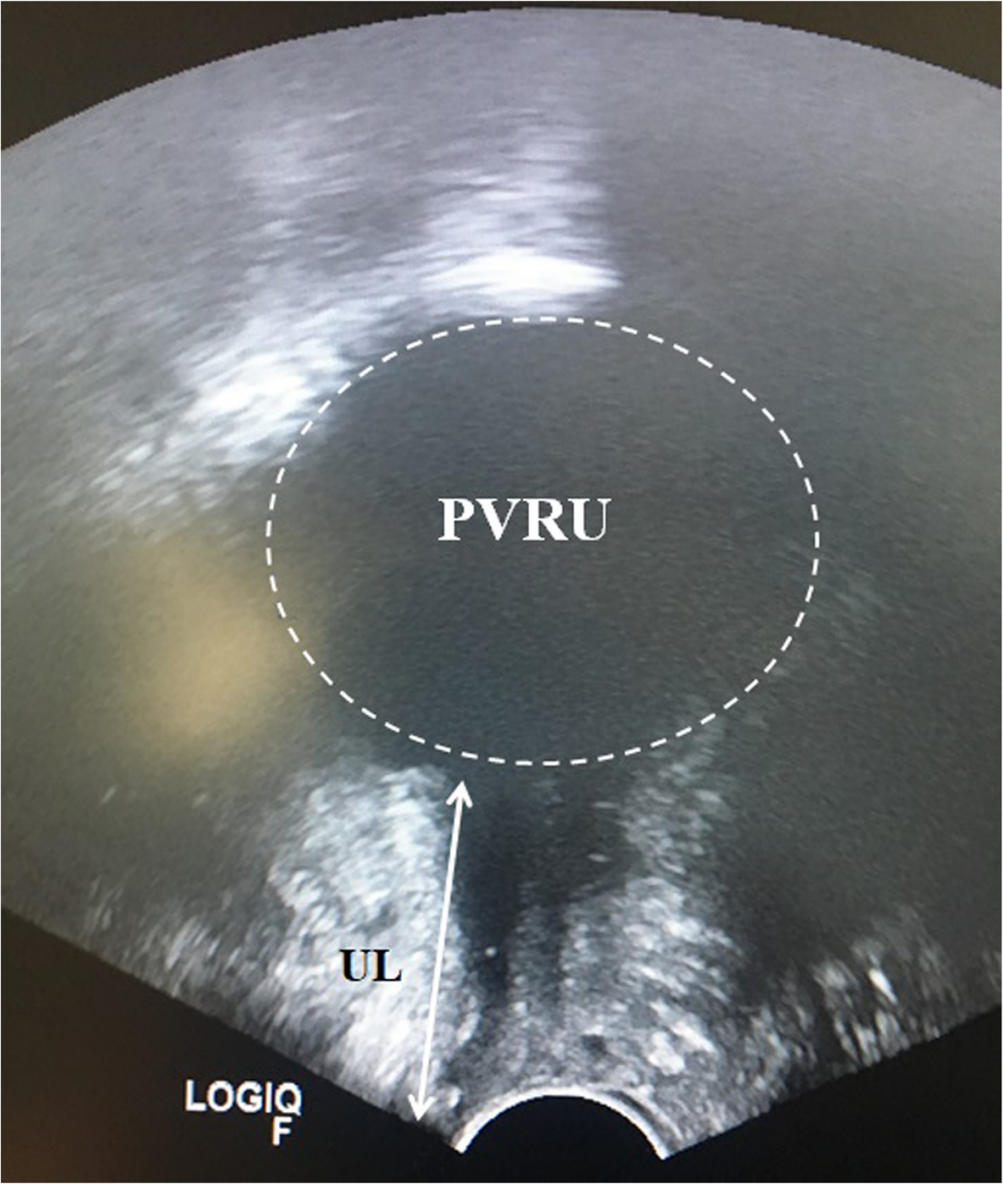
Vaginal ultrasound scan image of the urinary bladder after an attempt to completely empty the bladder. The examination was carried out with a LOGIQ S7^®^ ultrasound machine (GE Healthcare, Chicago, IL, USA). PVRU represents the post-void residual urine volume (hyperechoic), inside the urinary bladder (*dashed white line*), while UR is the urethral length (*arrow* - 3.66 cm). *PVRU* post-void residual urine

**Fig. 2:**
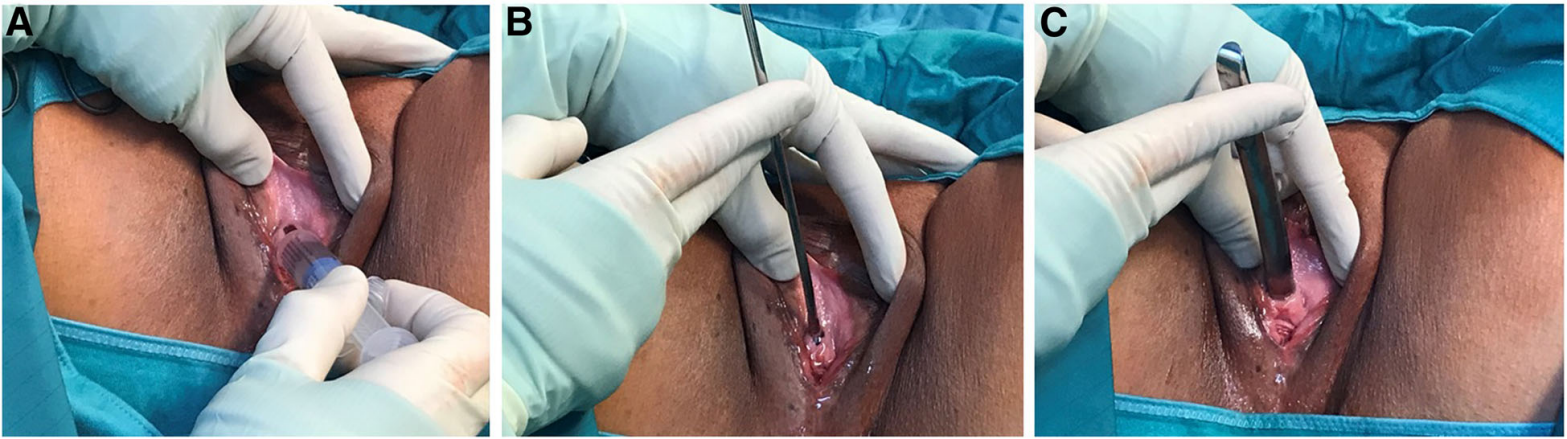
The dilatation process was initiated by lidocaine-containing jelly insertion (**a**), and was followed by the administration of rigid metallic Hegar sticks penetrating the external and the internal os of the urethra (**b**). Urethral length was assessed preoperatively with a vaginal ultrasound scan. The step-by-step dilatation was carried out until Ch39 (Hegar 13 mm – **c**)

**Fig. 3:**
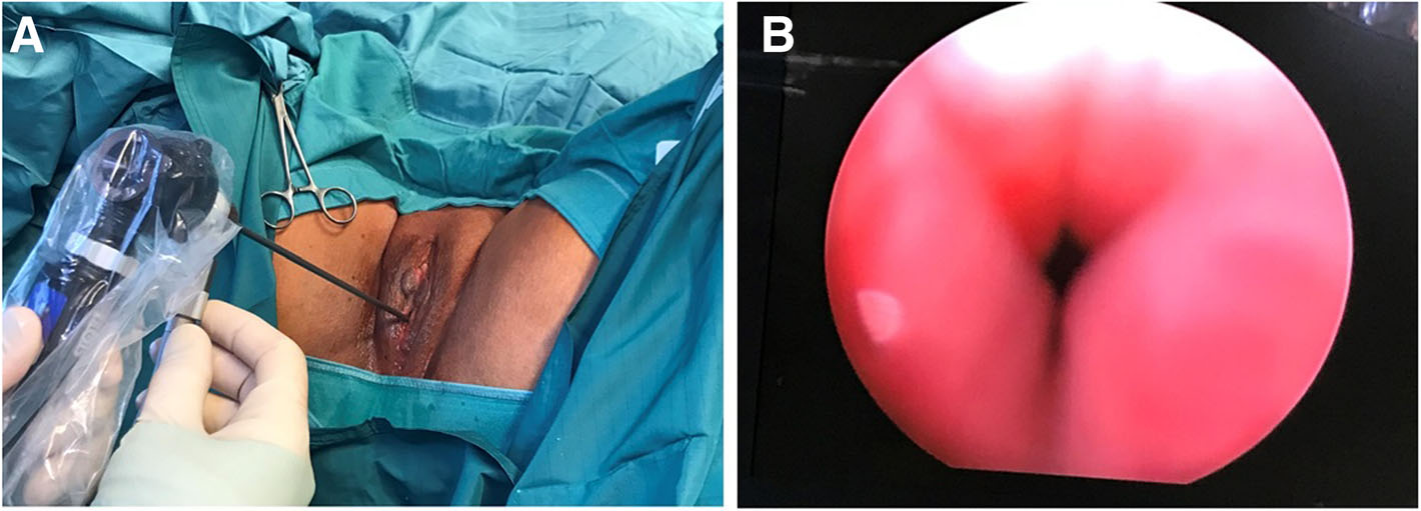
Intraoperative rigid uretrocystoscopy was carried out before and after the dilatation process (**a**). Significant stricture of the midurathral segment was visualized (**b**)

A 79-year-old Caucasian woman reported for an outpatient consultation, presenting with urgency (12–15 times/day), nocturia (3 times/night), inability to fully empty her bladder, and reoccurring UTIs (three times in the past 3 months). Our patient was declared cardiopulmonary stabile with blood pressure of 130/90 mmHg and a pulse of 67 beats/min. A detailed medical history revealed a multidrug regimen (bisoprolol 5 mg, candesartan cilexetil 16 mg, lercanidipin hydrochlorid 10 mg) controlled her hypertension for 15 years with the use of no diuretics, and the presence of open-angle glaucoma, which contraindicated the use of anticholinergic therapy. She took no other medications on a regular basis. Our patient had had three deliveries, including one vaginal birth and two cesarean deliveries. Previous operations included the following: benign left ovarian cystectomy, right nephrectomy after a vehicle accident, abdominal herniotomy, and anal abscess extirpation. Our patient was married, a retired elementary school teacher, with a history of no regular alcohol or drug consumption or smoking. Any allergy was not known. A blood test revealed normal liver and kidney function, and no sign of generalized infection (glutamic oxaloacetic transaminase [GOT]: 40 U/L, glutamic oxaloacetic transaminase [GPT]: 25 U/L, gamma-glutamyl-transpeptidase [GGT]: 43 U/L, amylase: 56 U/L, alkaline phosphatase: 91 U/L, lactate dehydrogenase [LDH]: 180/U/L, creatinine: 0.93 mg/dL, sodium [NA]: 138 mmol/L, potassium [K]: 4.1 mmol/L, calcium [Ca] 2.52 mmol/L, haemoglobin 13.9 g/dL, leukocytes: 6.6/nL, thrombocytes: 301/nL, erythrocytes: 4.8/pL, C-reactive protein [CRP]: 23. mg/L). On admission, a urine dipstick tested positive for nitrite, leukocyte esterase, and blood, with alkaline pH (8.5). Microbiology revealed ongoing *Escherichia coli* UTI, which was treated with oral cefuroxim 2 × 250 mg for 7 days. On urogynecological physical examination, we found no signs of urogenital prolapse (pelvic organ prolapse quantification [POP-Q] scores Aa: − 3, Ap: − 3, Ba: −3, Bp: −3, C: −7, GH: 2, Pb: 2, TVL: 9, D: −8), vaginitis, and vaginal or cervical erosion. Pelvic ultrasound confirmed no altered pelvic anatomy, with an atrophic 5.18 × 2.8 × 2.4 cm-sized uterus, with an endometrial thickness of 3 mm, sharp endomyometrial borders, with no sign of adnexal pathology, and no presence of free abdominal fluid. The introitus ultrasound found the urethral length to be 3.66 cm, with a significant visible stenosis at the middle segment of the urethra (0.4 mm). During catheterization, 150 mL post-void residual urine was detected (Fig. 1). A neurologic examination included the assessment of the tone, the strength, and the coordinated movements of the lower extremities, especially focusing on the abduction and the spreading of the toes, proving intact S3 innervation. The bulbocavernosus reflex and the anal sphincter reflexes were also found to be normal (intact S2˗S4 innervation). The preoperative urodynamic examination resulted in detrusor pressure at maximum flow ranges of 40 cm H_2_O with maximum flow rates (Q_max_) of less than 10 mL/s. After the informed consent of the patient, we carried out urethral dilation with ascending Hegar sticks ranging from 3 to 13 mm (Ch9 to Ch39) in diameter in general narcosis. Before each dilatator insertion, lidocaine jelly was administered transurethraly (Fig. 2). All metallic dilatators were inserted 4 cm deep in the long axis of the urethra, and then gently pulled saggitally downward till they met resistance. Intraoperative cefuroxime 1500 mg, and metronidazole 500 mg antibiotic prophylaxis was given to our patient to avoid postoperative uretrocystitis developing. Before and after the dilatation, cysto-urethroscopy was carried out, to confirm preoperative urethral stenosis, and no urethra or bladder injury postoperatively (Fig. 3). Six months after our patient’s surgery, she was reported her to be symptomless, continent, and the post-void residual urine volume was found to be completely eliminated, with no sign of UTI.

## Discussion

Here, we present a case of a patient with female urethral stricture (FUS) with storage and urinary symptoms (150 mL post-void residual urine preoperatively), which was successfully treated with extreme urethral dilation (Ch39). Urethral dilation is the benchmark of the management of FUS, however, available data on the method in the current literature is limited. Although the majority of available literature describes female urethral dilation until Ch 30 (10 mm) [4], based on our unpublished results and observations, this was found to be less effective, therefore we decided to proceed further with the dilation. The advantage of this method is that it is easy to carry out, and has been reported to have low intra-and postoperative complication rate, and can be performed in a 1-day surgery setting with low medical costs. The disadvantage of the approach includes potential complications during the intervention, such as urethral microrupture, which can lead to a UTI, therefore we suggest intraoperative combined antibiotic prophylaxis. We also recommend a gentle saggital downward push of the dilatators during penetrance of the urethra in order to ease and relax the pubourethral ligament, as described by Petros and Ulmsted [5], and to facilitate the urine outflow. A weak point of the method is that limited information is currently available on the effectiveness rate. While Romman *et al.* reported 51% success rate, Smith *et al.* published 57% success rate during follow-up of 21 months [6, 7]. Other surgical options include vaginal, or labial flaps, and also vaginal and oral mucosa grafts [8]. Vaginal flaps first described in 1989 by Blavas *et al*. [9]. These techniques use “U” or “C-shaped” vaginal flaps to reconstruct the urethra, and were reported to have a success rate of 80–100% [10, 11]. Another therapy option would be vaginal graft urethroplasty, which has, based on the limited available data, a success rate ranging from 75 to 100% during a mean follow-up between 15 and 24 months [12–14]. Use of buccal or lingual mucosa grafts are also looking to be a promising surgical approach to treat FUS, but missing long-term data, and studies with a large number of participants are currently missing.

## Conclusions

According to the literature, urethral dilatation remains the cornerstone of PBNO treatment, despite its success rate. We believe that urethral dilatation should not be terminated at Ch30, and the urethra should be and can be further dilated until 13 mm. Since we only present a single case, long-term studies with a large number of participants are necessary to confirm our theory, as well as a correlation analysis between the post-void urine volume and the gauge of the dilation.

## Abbreviations

Ch: Charrier (1 mm = 3 Ch)
FUS: Female urethral stricture
LUTS: Lower urinary tract symptom
PBNO: Primary bladder neck obstruction
POP-Q: Pelvic organ prolapse quantification
Q_max_: Maximum flow rate
UTI: Urinary tract infection
